# 

**Published:** 1916-12

**Authors:** 


### The Editor's Chat.

Last month the printers crowded out Dr. John S. Turner's able contribution on "Delinquency from a Psychic Standpoint," but this month it will be found on the front page. It is one of the strongest and most interesting articles that has appeared in the JOURNAL for months and should have a careful reading.

By this time every old reader of the JOURNAL has come to know Dr. Henry S. Harrower for his very excellent presentation of "The Diagnosis of the Internal Secretory Diseases," the seventh of which appears in this issue. This paper deals with "Diseases of the Thymus," and will be found up to the usual Harrower standard.

Through the courtesy of Dr. James A. Hill in calling our attention to it and through the kindness of Dr. C. U. Patterson, our readers are this month given a treat in a paper from Dr. Patterson on "Blood Pressure as an Aid to Diagnosis," In sending the paper Dr. Hill said: "This paper has created as much discussion in our society as any paper that has been read for quite a while, and for this reason I believe that other men throughout the State and elsewhere will be glad to read such a paper."

Physicians everywhere will take much interest in reading an excellent article this month from Dr. Arthur H. Merritt of New York on "Mouth Infections and Their Systemic Effect." Dr. Merritt is an authority, as his paper shows, and he writes with that interest which holds readers to the subject. In January, Dr. C. M. McCauley's third paper will appear, his subject being "Dental Caries."

Lack of space on this page prevents extended notice of the pellagra department which is up to its usual standard. Our readers are strong in the commendations of these efforts to keep them informed upon the new problems constantly arising in the medical profession which can be done only with the assistance of men eminent as specialists in the departments they conduct.

In keeping with our promise faithfully made to our readers we are doing our best to make the TEXAS MEDICAL JOURNAL better with every issue. We think those of you who were our subscribers a year ago will agree that the promise has been kept. With the help of the physicians of the country, 1917 is going to be made the best year in all the life of the JOURNAL. Will you be one to aid just a little?

The JOURNAL will close the year with three times as many subscribers as it had at the beginning of the year. We intend to try to keep up a proportionate growth during 1917, and can do it if every friend will continue as loyal as in the past.

